# Multiple Synchronous Mucosa-Associated Lymphoid Lymphomas Involving in the Stomach, Duodenum, Ileum, and Sigmoid

**DOI:** 10.3390/diagnostics12123150

**Published:** 2022-12-13

**Authors:** Chun-Wei Chen, Yang-Yuan Chen, Yung-Fang Chen

**Affiliations:** 1 Division of Gastroenterology, Yuan Lin Christian Hospital, Yuanlin City 510, Taiwan; 2 Endoscopic Center, Changhua Christian Hospital, 3 Lane 138 Tai-An 2nd Street, Changhua City 500, Taiwan; 3 Yuan Lin Branch, Department of Hospitality Management, MingDao University, Changhua City 523, Taiwan; 4 Division of Radiology, China Medical University, China Medical University Hospital, Taichung City 404, Taiwan

**Keywords:** gastric MALToma, duodenal MALToma, ileal MALToma, sigmoidal MALToma

## Abstract

Gastric MALToma is an extra-nodal marginal-type B-cell lymphoma. MALToma may occur secondary to chronic inflammation and autoimmunity. The most common gastrointestinal (GI) site of MALToma is the stomach, with approximately 50% of lesions occurring there. Synchronous upper and lower GI MALTomas rarely occur, with few cases reported. We present the case of a 67-year-old patient who presented asymptomatic and was found to have synchronous multifocal upper and lower GI MALTomas in the stomach, duodenum, terminal ileum, and sigmoid, which did not respond to H. Pylori eradication therapy.

This work studies a 67-year-old man who had a helicobacter (HP)-related gastric MALToma 17 years ago. He received HP eradication at the time and made a complete recovery. He had two upper endoscopies, two and five years later; the endoscopic findings showed only the presence of scar ulcers without any MALToma at that time. The endoscopy revealed a gastric ulcer over the posterior wall of a high body, hyperemia, and mild nodularity over the duodenal bulb (Figure 1A,B). The body and antrum of the stomach and beyond the duodenal bulb area showed no hyperemia or ulcer changes. The MALToma was a suspicious recurrence. For MALToma staging, we performed an abdominal CT scan and noted two segments of intestinal wall in the ileum and sigmoidal colon (Figure 2C,D), and the normal gastric and duodenal wall (Figure 2A,B). Subsequently, double-balloon enteroscopy revealed that the sigmoidal colonic mucosa presented hyperemia change and the ileal wall presented multiple tiny diffuse granules (Figure 1C,D). Physical examination yielded no remarkable findings in the abdomen, and laboratory testing revealed no changes in the complete blood cell count and biochemistry. A biopsy from the stomach, duodenum, ileum, and sigmoidal colon were performed thereafter.

The pathological analysis of the biopsy samples from four gastrointestinal areas revealed monotonous lymphocyte infiltration in the lamina propia with a lymphoepithelial lesion and without Helicobacter infection in stomach (Figure 3A), and atypical lymphocyte infiltration in the lamina propia with a lymphoepithelial lesions in the duodenum, ileum, and sigmoidal colon (Figure 3B–D). Subsequent immunohistochemical (IHC) analysis showed a lymphoid infiltrate with numerous CD20+ B cells. BCL-2 was positive, whilst CD10 and BCL-6 were negative. There was no light chain restriction. We did not perform an IgH rearrangement test or API2MALT1 tests. The MALToma was diagnosed with synchronous involvement in the stomach, duodenum, ileum, and sigmoidal colon. The patient received HP eradication therapy again before chemotherapy, even though findings of the HP study were negative. The lesions including all four areas did not response to HP eradication on this occasion. The patient was thus administered rituximab, cyclophosphamide, doxorubicin, vincristine, prednisolone (R-CHOP) treatment. The follow-up abdominal CT scan and endoscopy indicated complete resolution.

Gastric MALToma is an extra-nodal marginal-type B-cell lymphoma. MALToma may occur secondary to chronic inflammation and autoimmunity. The most common GI site of MALToma is the stomach, with approximately 50% of lesions occurring there, followed by the small bowel, ileocecal region, colon, and rectum. Non-GI sites of MALToma include the lungs, salivary glands, thyroid gland, skin, and eyes. The presence of synchronous upper and lower GI MALTomas appears to be an extremely rare occurrence, with only a few cases reported [1,2,3]. This is the first report involving four separate synchronous MALTomas in the upper and lower GI tract.

Most patients with gastric MALToma are asymptomatic or complain of nonspecific gastrointestinal symptoms [4]. The endoscopic features of gastric MALToma are diverse and nonspecific. Gastric MALTomas have a classification as follows: (1) ulcerative: single to multiple ulcerations; (2) exophytic: a single mass or polypoid aspect; (3) hypertrophic: enlarged folds and a nodular pattern; (4) petechial haemorrhage: several petechiae at mucosa; (5) hyperemic mucosa: reddish mucosa; and (6) mixed: two or more combinations of the above-mentioned patterns. Ulceration is the most common presentation [5]. However, Ishikawa et. al. reported that superficial appearance is the most common endoscopic presentation in a recent report, accounting for approximately 70–80% of all cases [6]. Colonic MALToma was described as a single polypoid, ulceration, or hyperemic mucosa lesions in another endoscopic study [7,8,9]. Our patient was also asymptomatic. His endoscopy results indicated an ulcer in the stomach, hyperemic mucosa in the bulb of the duodenum and sigmoidal colon, and multiple small nodules in the ileum.

The four involved sites of the GI tract in the present case are distant from each other, therefore, the MALToma are likely developing independently. The treatment of concurrent upper and lower GI tract MALTomas involves R-CHOP chemotherapy, and our patient exhibited a complete response. The detection of synchronous lesions will obviously affect their long-term prognosis, as synchronous lesions will likely require far more aggressive therapy than isolated gastric MALTomas [10].

## Figures and Tables

**Figure 1:**
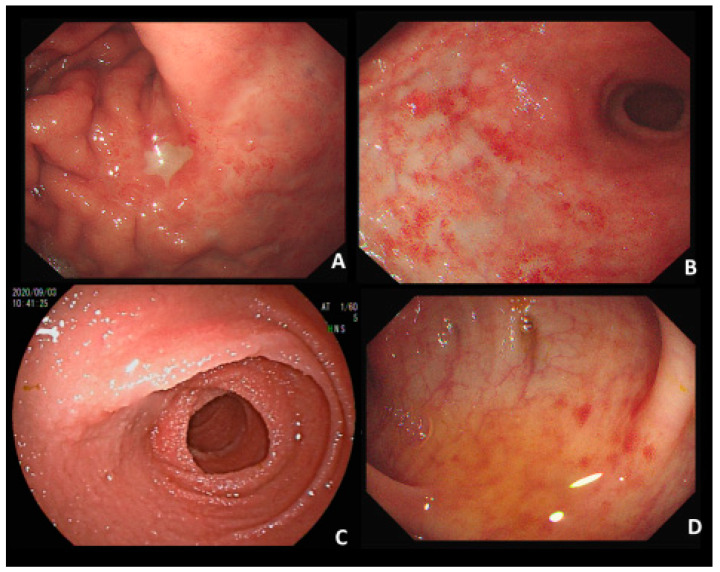
The endoscopy revealed MALToma in the stomach (**A**), duodenum (**B**), terminal ileum (**C**), and sigmoid (**D**).

**Figure 2:**
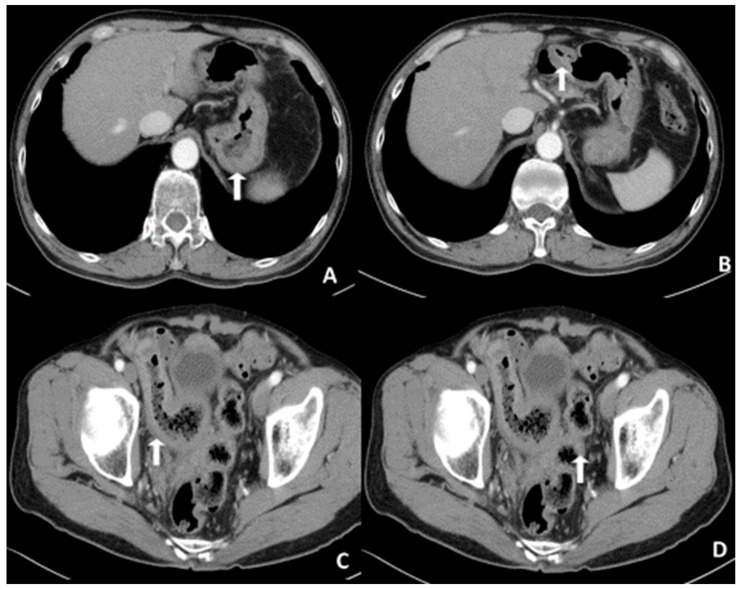
The abdominal CT scan revealed MALToma in the stomach (**A**), duodenum (**B**), terminal ileum (**C**), and sigmoid (**D**).

**Figure 3:**
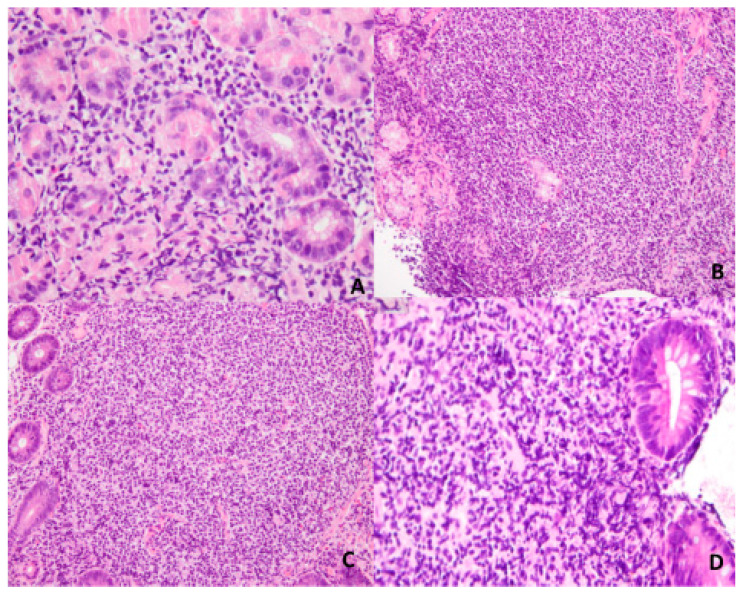
The pathology revealed MALToma in the stomach (**A**), duodenum (**B**), terminal ileum (**C**), and sigmoid (**D**).

## Data Availability

Not applicable.
